# Facile isolation and analysis of sporopollenin exine from bee pollen

**DOI:** 10.1038/s41598-021-87619-8

**Published:** 2021-05-11

**Authors:** Kristóf Hegedüs, Csaba Fehér, István Jalsovszky, Zoltán Kristóf, János Rohonczy, Elemér Vass, Attila Farkas, Tamás Csizmadia, Gernot Friedbacher, Peter Hantz

**Affiliations:** 1 Institute of Organic Chemistry, Research Centre for Natural Sciences, Magyar Tudósok Körútja 2, Budapest, 1117 Hungary; 2 Department of Applied Biotechnology and Food Science, Budapest University of Technology and Economics, Szent Gellért tér 4, Budapest, 1111 Hungary; 3 Department of Organic Chemistry, Eötvös Loránd University, Pázmány Péter sétány 1/A, Budapest, 1117 Hungary; 4 Department of Plant Anatomy, Eötvös Loránd University, Pázmány Péter sétány 1/A, Budapest, 1117 Hungary; 5 Department of Inorganic Chemistry, Eötvös Loránd University, Pázmány Péter sétány 1/A, Budapest, 1117 Hungary; 6 Department of Organic Chemistry and Technology, Budapest University of Technology and Economics, Budafoki út 8, Budapest, 1111 Hungary; 7 Department of Anatomy, Cell and Developmental Biology, Eötvös Loránd University, Pázmány Péter sétány 1/C, Budapest, 1117 Hungary; 8 Institute of Chemical Technologies and Analytics, Vienna University of Technology, Getreidemarkt 9, 1060 Wien, Austria; 9 Fibervar Llc., Str. Bolintineanu Nr. 20, 400062 Cluj/Kolozsvár, Romania; 10 Centre for Ecological Research, Karolina út 29, Budapest, 1113 Hungary

**Keywords:** Chemical engineering, Biomaterials, Plant sciences, Chemistry, Materials science

## Abstract

We present facile methods to obtain purified sporopollenin exine capsules, and provide mass balances for classical and novel purification procedures. An ionic liquid, tetrabutyl phosphonium hydroxide turned out to be the most effective in removing the intine wall. The sporopollenin capsules were investigated by fluorescent microscopy, AFM, solid-state NMR and infrared Raman spectroscopy. The latter two methods showed that sunflower and rape exines have different proportions of O-aliphatic and aromatic constituents. Purified exine capsules were coated with functionalized fluorophores. The procedures presented in this paper could contribute to further spread of the applications of this hollow, and chemically highly resistant material.

## Introduction

Pollen grains are the male gametophytes of seed plants. They are produced by the anther of the flowering plants, and transferred to the stigma by wind or by insect vectors. The grains, having a diameter between 3 and 250 µm, possess a complex microstructure^1–5^. On their periphery one finds a fatty material, the pollen coat (its variations are referred to as the pollenkitt or tryphine)^6–9^. This stands on the sculptured exine shell, consisting of sporopollenin, which is one of the most chemically inert biopolymers^1,2,10–15^. In monocot species the exine usually has one, while in eudicots three apertures (regions where the exine shell is thin or hollow)^2^. Below the exine, one finds the intine wall, a closed, cellulose-rich layer surrounding the pollen surface underneath the exine shell^16–18^. Within this intine wall, there are two or three cells (a generative cell or two sperm cells inside the large vegetative cell) accumulating lipids and polysaccharides as storage materials^7,19–22^.

Note that the term “bee pollen” denotes pollen gathered by honeybees. During the collection, pollen grains are appended to the loads (pellets) in the corbiculae of workers. The beekeeper harvests the loads by an accessory placed to the entrance of the hive. The millimeter-sized pollen loads contain sugar-rich nectar as well, disgorged by the worker while forming the loads^23–26^. In order to enable storage of loads at room temperature, they can be dehydrated at 30–40 °C, until their dry matter content reaches about 95%.

There is a growing interest in obtaining purified sporopollenin exine shells. Several attempts were made to apply these hollow microscopic capsules for controlled drug release^27–29^ and taste masking^30^. There is some evidence that microencapsulation in exine shells enhances the bioavailability of certain fatty acids^31^. Functionalized exine fragments were tested as heavy metal scavengers^32,33^. Emulsions were stabilized by purified exine capsules^34^. Sporopollenin exine grains have also been used as a support for enzymes^35^ and ionic liquids^36^. Sunflower exine capsules with partial platinum coating enabled the catalytic decomposition of hydrogen peroxide, resulting in bubble-propelled micromotors^37^.

Pollen grains have been used to probe active transport of microparticles through the intestinal mucosa, that is, from the intestinal content into the bloodstream. Although this phenomenon, also referred to as *persorption*, has already been reported by a series of studies^38–40^, its existence is not widely accepted. There is, however, a substantial number of publications reporting the dissolution of exine particles in the bloodstream^41–43^. Note that it is a challenge to detect exine shells in the blood or in the plasma.

Mainstream approaches for obtaining purified sporopollenin exine shells comprise two work phases. The first one is devoted to remove fats and water-soluble carbohydrates; most of these are outside the exine shell and cement the millimeter-sized pollen load. The second procedure aims to dissolve the cellulose-rich intine wall, and to get rid of cellular debris^44^. As we will discuss in more detail, dissolution was pioneered by Fritz Zetsche in the first decades of the 20st century, while alternative methods are being developed up to the present.

The first phase of the purification is implemented by stirring and filtering, or by performing Soxhlet extractions^45,46^. For this purpose, it is advantageous to use a series of solvents with increasing polarity^47,48^. Washing the pollen loads with a mixture of solvents^49,50^ was also applied. Mono- and disaccharides can be solubilized and removed by washes using polar solvents like water^51^, while apolar solvents remove fatty and waxy ingredients. Aqueous detergents were used for extracting pollen coat proteins^29,46,52,53^ and detergent-insoluble microdomains of pollen tubes^54^. Note that it is hard to estimate the extent to which these solvents affect the protoplasm protected by the intine wall^7,19,55^.

For the second phase of the purification, that is, for dissolving the cellulosic intine wall, Zetsche developed protocols employing so-called alkaline lysis and acetolysis. These methods consist of immersing pollen grains for 6 h and at elevated temperatures in a strong base like 5% potassium hydroxide solution^56^, or soaking them for 7 days in a strong acid like 85% phosphoric acid^57^. Subsequently, several combinations and refinements of Zetsche's methods have also been proposed^58–60^.

Another method uses a mix of acetic anhydride and sulfuric acid to obtain purified exine shells, even without any initial cleaning^61^. Anhydrous hydrogen fluoride has also been applied for intine wall removal^62^. Recently, certain ionic liquids, like tetrabutyl-phosphoniumhydroxide (TBPH) have been suggested to use in order to obtain purified sporopollenin exine shells^63^.

More gentle methods, namely enzymatic cleanings, have been elaborated as well. A cocktail of two enzymes was reported to degrade the intine wall in 3 days at 30 °C^50,64^, and in 16 h at 37 °C^1^. A method employing a series of 5 to 10 different enzymes hydrolyzing polysaccharides was also reported to be successful^49,65^.

Present purification techniques have certain drawbacks. Most of them employ noxious compounds, while others might even damage certain types of exine^66^. Enzymatic methods could also be optimized in several respects like pretreatments and the composition of the cocktails used.

For none of the purification methods were the mass balances determined, which would allow comparison of their efficacy. In this paper, we provide mass balances for a series of classical and novel cleaning protocols, and we refine several methods to obtain purified bee pollen exine shells.

Sporopollenin exine derivatization has a major importance in several fields of science. However, due to its extreme inertness, this is a demanding task. In this publication, we present a protocol to coat exine shells with functionalized fluorophores as model compounds for further studies. We implemented a selective amine reactive linking method, using the widely available FITC (Fluorescein isothiocyanate) and RBITC (Rhodamine B isothiocyanate) to visualize the functionalization of the exine surface.

## Results and discussion

**Diagram 1:**
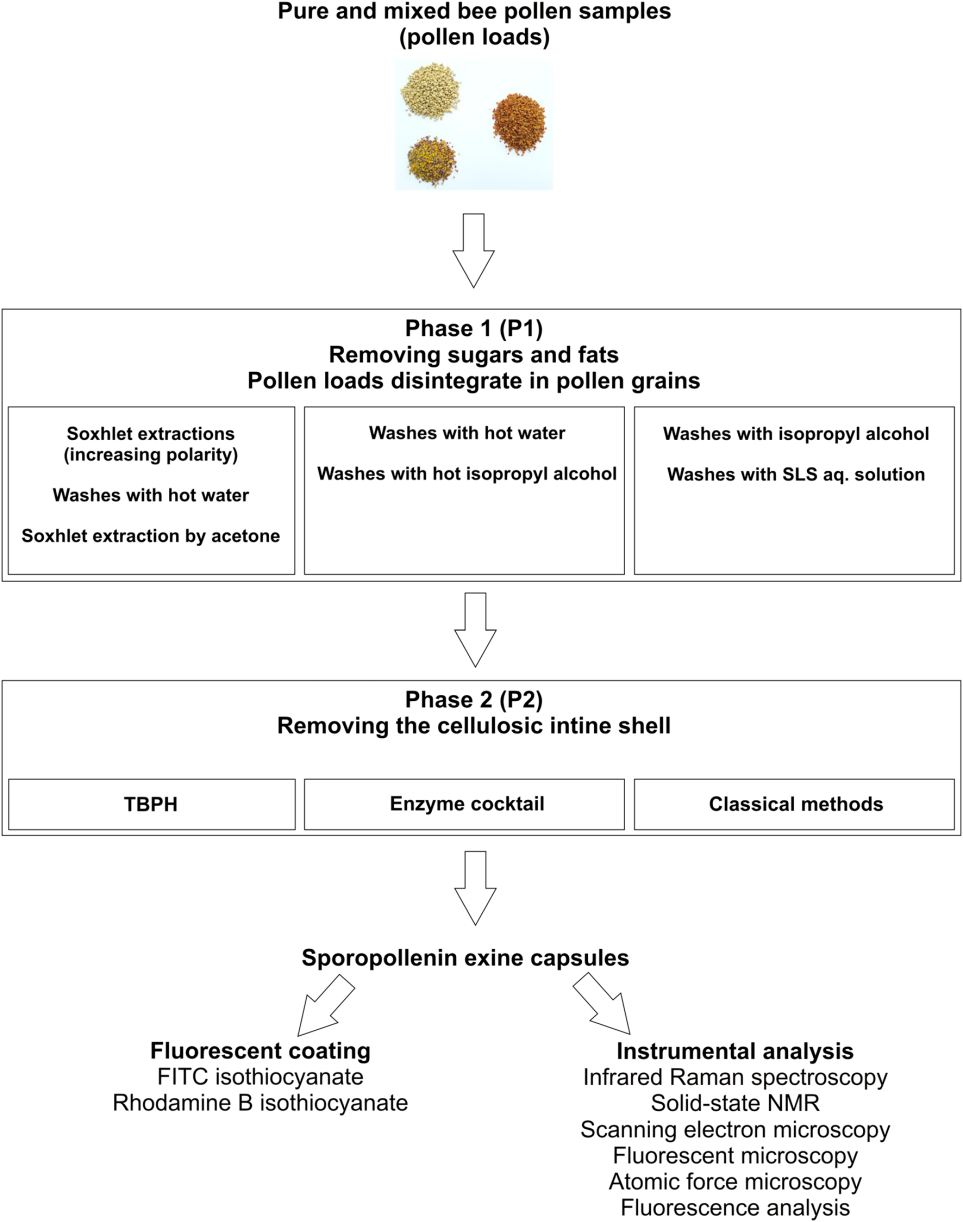
Sketch of the pollen purification procedure, subsequent instrumental analysis and chemical coating.

The workflow followed in this study is summarized in Diagram 1.

The bee pollen samples were purified by a two-phase protocol. The first phase (referred to as P1) removed soluble fats and carbohydrates, while the second one (referred to as P2) aimed to dissolve the cellulosic intine wall with the cells in its interior.

Subseqently the best P1-purified grains were investigated by fluorescence microscopy, while the best P1 + P2-purified exine shells were examined by a series of microscopy and instrumental analytical methods. Fluorescent and electron microscopy served for determining the presence and absence of the intine wall, while infrared Raman spectroscopy and solid-state NMR for deciphering some aspects of sporopollenin composition. Moreover, cleaned sunflower and rape sporopollenin shells were coated by fluorescent dyes.

### First purification phase (P1): removal of the pollenkitt and the sugar-rich material cementing the pollen loads

#### Soxhlet extractions followed by an aqueous and acetone wash

Table 1 presents how the dry mass of the bee pollen samples altered along the Soxhlet extractions followed by a final aqueous and acetone wash. Dry matter contents of the starting samples were 96% (mixed), 96.6% (rape) and 96.6% (sunflower); the percentages of the residues at different steps were computed taking into consideration the initial dry matter content.

**Table 1:** Alteration of the dry mass of bee pollen samples following Soxhlet extractions and a final aqueous and acetone wash, compared to the initial dry mass content.

	Mixed		Rape		Sunflower	
	Extracted %	Residue %	Extracted %	Residue %	Extracted %	Residue %
Chloroform	5.36	110.89	1.14	108.59	4.34	100.88
IPA	45.88	54.27	35.72	65.27	49.43	47.93
Methanol	15.88	33.44	23.03	36.33	15.94	25.62
Water	7.5	25.94	6.78	29.55	6.0	19.61
Acetone	–	Unchanged	–	Unchanged	–	Unchanged

Most of the dry matter samples were removed by the first polar solvent (here isopropyl alcohol, IPA). Surprisingly, following the extraction with the first, nonpolar solvent, the mass of the pollen dried at 5 mbar at 60 °C increased. The reason for this could be the adsorption of these solvents into the pollen grains. We also mention that the cleansing with acetone performed after the aqueous wash did not remove a measurable amount of material. Note that due to their hygroscopic nature, the samples may absorb some humidity if removed from the desiccator.

#### Aqueous washes followed by isopropyl alcohol extraction

P1-purification of bee pollen samples were also implemented by a wash with water followed by a wash with IPA. The aqueous washes at different temperatures lead to similar results, except for the rape pollen, where, surprisingly, the wash with room temperature water was more efficient (Table 2). We speculate that some structural changes could occur within the rape pollen grains at elevated temperature which impede the purification process. The subsequent wash with IPA also removed a significant amount of soluble matter as well.

**Table 2:** Alteration of the dry mass of bee pollen samples following aqueous and IPA washes at different temperatures.

	Mixed residue %	Rape residue %	Sunflower residue %
Hot water	35.0	48.39	26.03
Hot IPA	26.95	33.1	19.61
RT water	34.79	37.73	28.10
RT IPA	27.95	26.8	21.17

Note that washes with isopropyl alcohol followed by 10% Sodium Lauryl Sulfate (SLS) aqueous solution followed by aqueous washes did not lead to better results, likely because SLS remained attached to the pollen structures. In the protocol using centrifugation instead of filtering, the losses during the centrifugation steps could not be avoided, therefore this procedure is inadequate for setting up mass balances.

We also mention that P1-purified bee pollen grains will change color, usually turning beige, light brown, or light green.

### Second purification phase (P2): removal of the intine wall and cellular debris

#### Classical procedures and purification by an ionic liquid, TBPH

**Figure 1:**
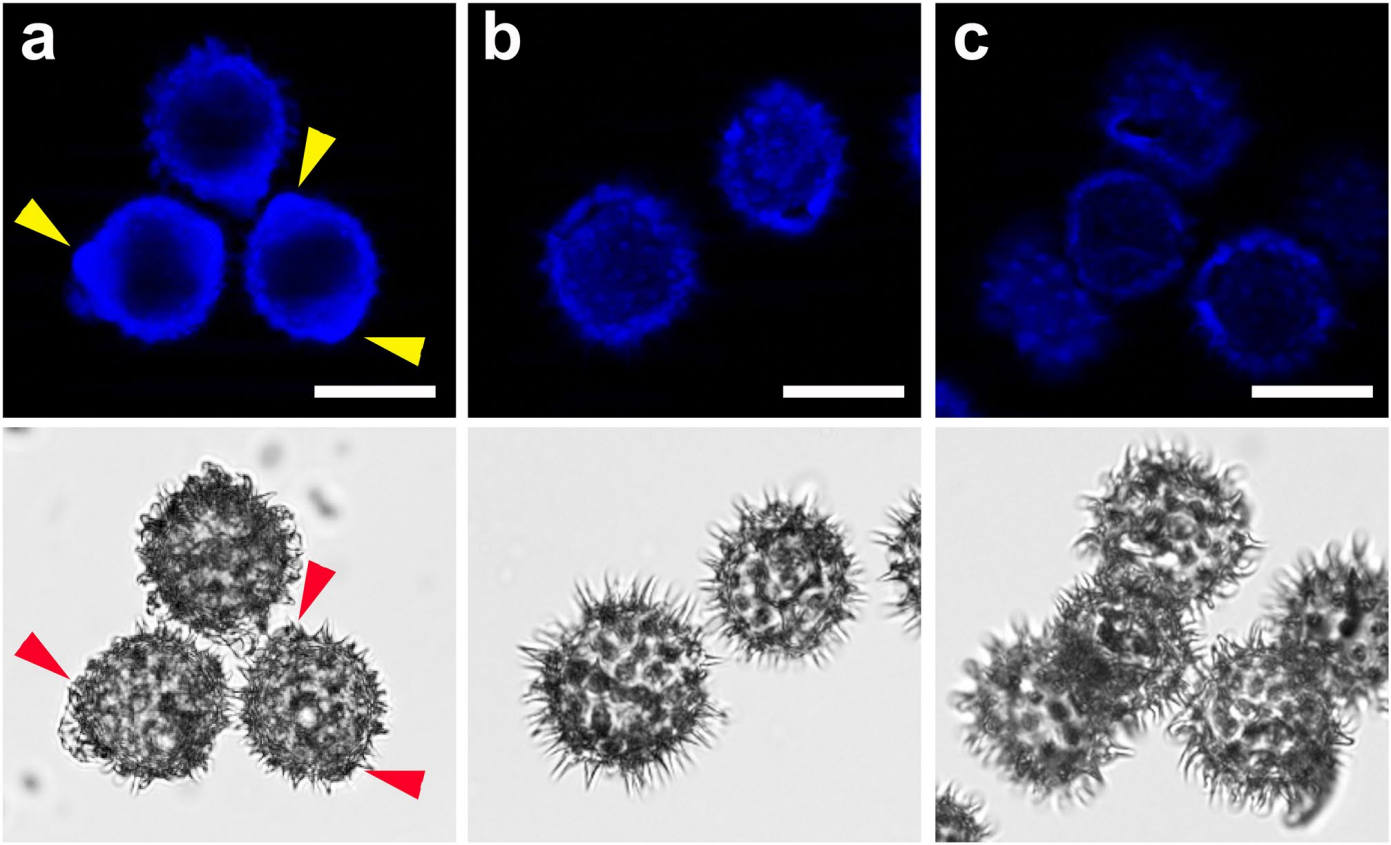

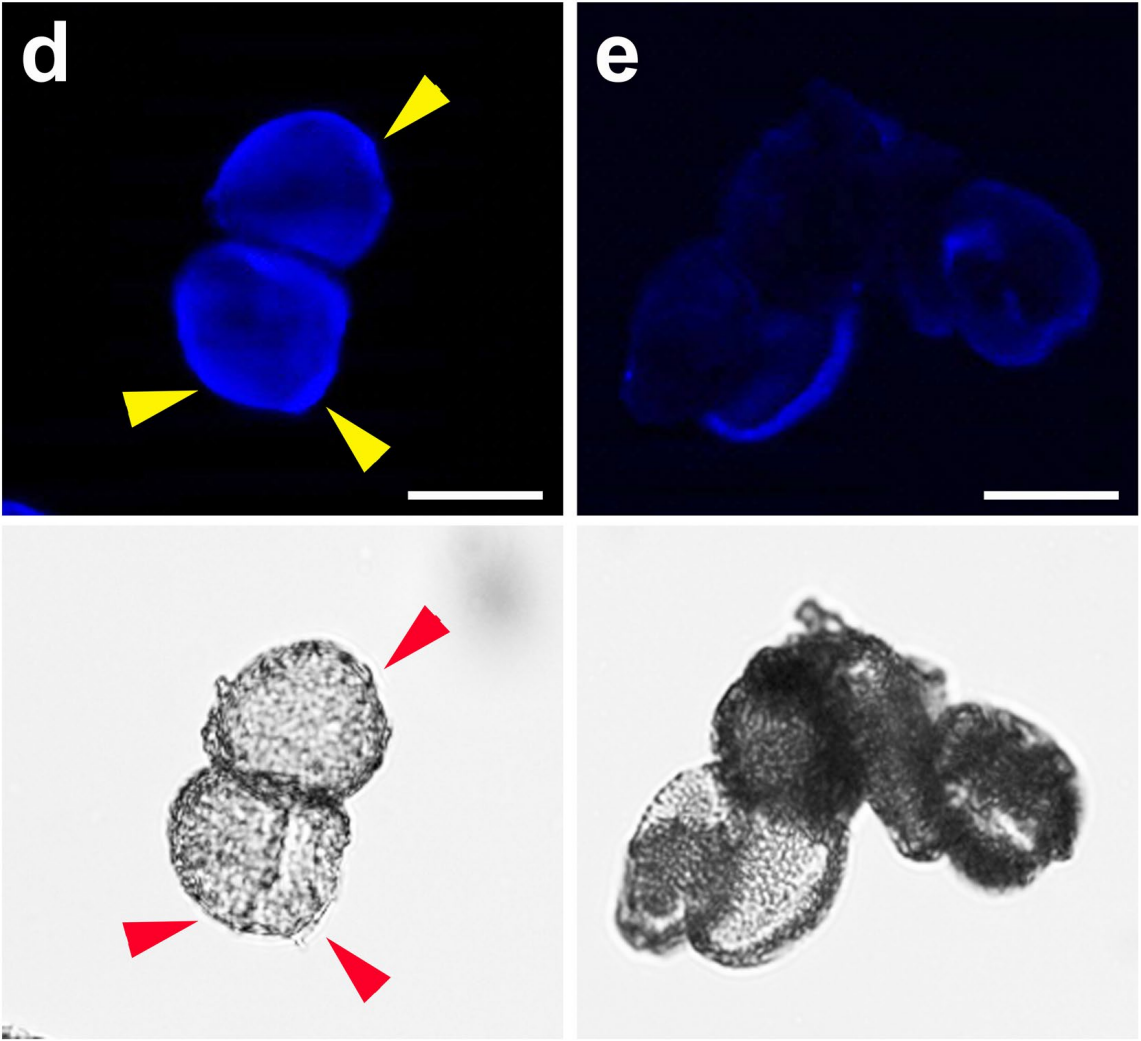
Fluorescence imaging of sunflower and rape pollen grains, with and without the intine wall. The samples were stained with calcofluor white. Pictures in the upper row were taken using mercury lamp excitation and a DAPI filter cube (see the Methods section), while the lower ones with brightfield imaging. Some exine apertures where the presence and absence of the intine can be best observed, are marked by yellow (on fluorescent image) and red (on brightfield image) arrowheads. Scale bars represent 40 μm. (**a**) Sunflower pollen grains P1-purified by Soxhlet extractions and an aqueous wash, without a P2-purification. Note the presence of the protruding intine portions. (**b**) Sunflower pollen grains P1-purified by Soxhlet extractions and an aqueous wash, and P2-purified by enzymatic treatment. Note the absence of the intine. (**c**) Sunflower pollen grains P1-purified by Soxhlet extractions and an aqueous wash, and P2-purified by TBPH. Note the absence of the intine. (**d**) Rape pollen grains P1-purified by Soxhlet extractions and an aqueous wash, without a P2-purification. (**e**) Rape pollen grains P1-purified by Soxhlet extractions and an aqueous wash, and P2-purified by TBPH. Note the absence of the intine.

Mass balances of the classical P2-purification procedures were roughly the same, except the one applying room temperature phosphoric acid, which had a lower efficacy (Table 3). However, there was a difference in affecting the fine spatial structure of the sporopollenin exine shells. Optical microscopy revealed that in case of purification by enzymatic digestion (Fig. 1b), TBPH (Fig. 1c, e) or H_3_PO_4_ (image not provided) the shells remain intact, while applying strong bases affected the main features of the exine structure (Supplementary Material Figure S2).

**Table 3:** Ratio of the dry matter content of bee pollen samples P1-purified by Soxhlet extractions and an aqueous was, before and after different P2 purifications (removal of the intine wall and the cellular debris).

	Mixed P2 residue %	Rape P2 residue %	Sunflower P2 residue %
Hot 10% aq. KOH	29	18	31
RT 10% aq. KOH	26	18	30
Hot H_3_PO_4_	35	15	28
RT H_3_PO_4_	84	68	38
TBPH	28	12	32

Classical protocols and a method using TBPH are compared.

#### Enzymatic digestion preceded by mild acidic pre-treatment

After performing a mild pretreatment with 2% w/w sulfuric acid, a mixture of Cellic Ctec2 and NS22118 enzymes was used to solubilize the intine wall. Incubation in acidic solutions represents an effective pretreatment for industrial-scale enzymatic digestion of lignocellulosic materials. Due to its low cost, diluted sulfuric acid is the most widely used agent for acidic pretreatment^67,68^. Cellic CTec2 is a commercial enzyme mixture with proved effectiveness on a wide variety of acidically pretreated lignocellulosic substances for the conversion of the carbohydrates into monosaccharides. Cellic CTec2 exhibits high cellobiohydrolase, endoglucanase, β-glucosidase and xylanase activities^69^. Supplementation of the cellulose-hydrolyzing cocktails by β-glucosidase activity (e.g. NS22118) is very often required to enhance the efficiency of the hydrolysis as β-glucosidases can act synergistically with the cellobiohydrolases and endoglucaneses^70^. Table 4 presents the mass balances of enzymatic digestion for different bee pollen samples and distinct P1 purification protocols.

**Table 4:** Average ratio of the dry matter content of bee pollen samples P1-purified by different methods, before and after enzymatic P2 purification (removal of the intine wall and the cellular debris).

Type of pollen	P1 purification protocol	P2 residue %
Mixed	Soxhlet extractions	38.5
	Hot aqueous and IPA washes	57.1
	10% SLS and IPA washes	60.4
Rape	Soxhlet extractions	28.9
	Hot aqueous and IPA washes	41.8
	10% SLS and IPA washes	49.5
Sunflower	Soxhlet extractions	38.4
	Hot aqueous and IPA washes	55.7
	10% SLS and IPA washes	57.3

Results of four experiments were averaged. The results are corrected with the losses occurring during the purification procedure. Losses were estimated by applying a similar purification protocol without using enzymes.

Note that the enzymatic digestion lead to the best results when the P1-purification was implemented by Soxhlet extractions. We speculate that the reason for this is rooted in the fact that this purification method reveals the intine wall most successfully.

### Instrumental analysis

#### Examining intine status by fluorescence microscopy

We compared pollen samples solely P1-purified (with an intact cellulosic intine wall), with the ones having the intine wall removed by P2-purification using TBPH or enzymatic treatment (subsequent to a P1-purification). All of the investigated samples were stained by calcofluor white, as described above. Fluorescence imaging clearly showed the presence or absence of intine portions protruding out through the apertures of the sporopollenin exine shell (Fig. 1).

Although the sporopollenin itself also shows some autofluorescence^71,72^, the smooth, protruding intine wall could clearly be distinguished (Fig. 1a). Moreover, this method turned out to be the least expensive and most reliable to determine whether the investigated protocol had worked (the majority of the intine wall and the cellular debris had been removed) or failed (the intine was still present or the sporopollenin exine structure was visibly damaged).

#### Examining pollen grain integrity and intine status by environmental scanning electron microscopy (ESEM)

**Figure 2:**
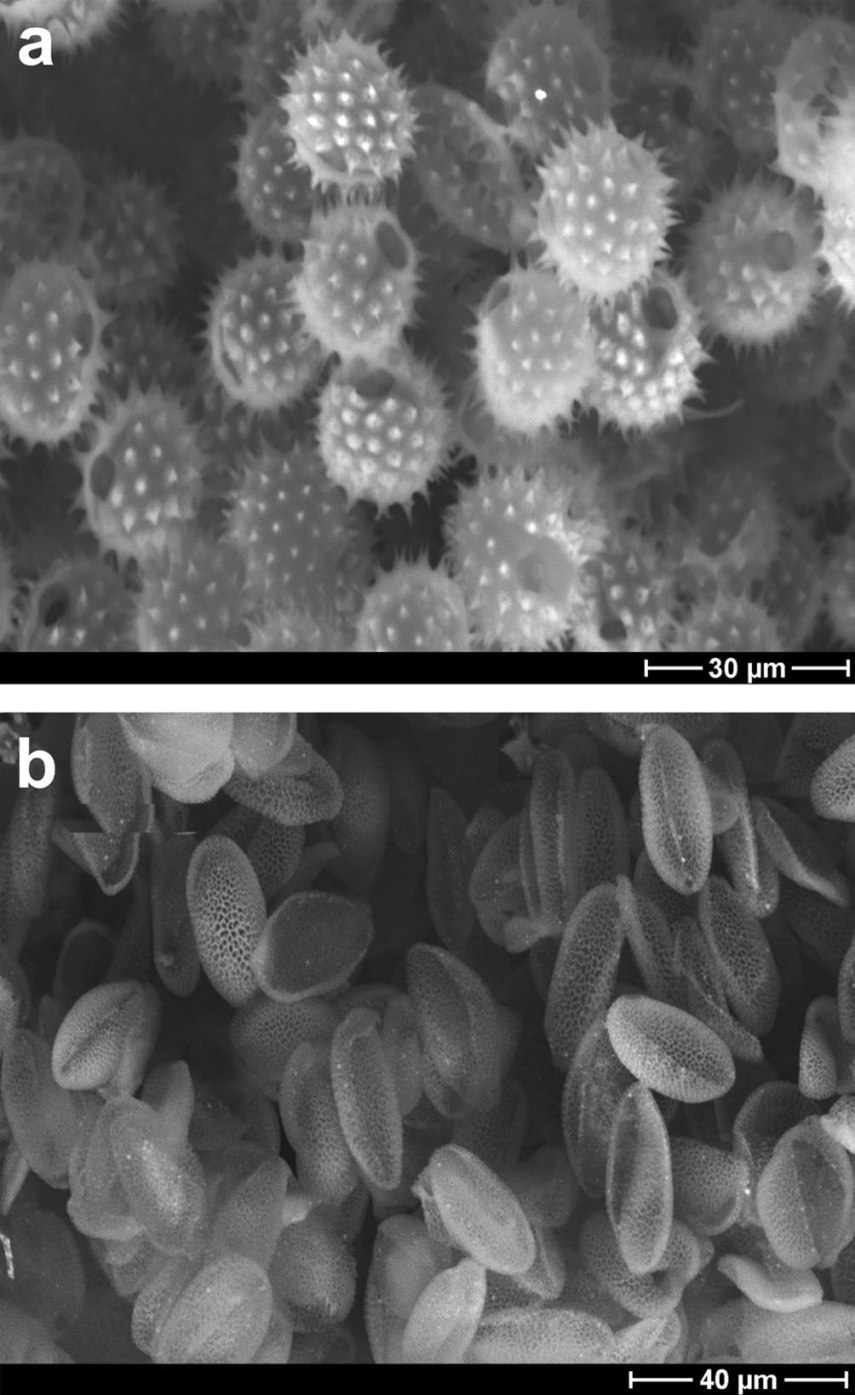
ESEM images of (**a**) Sunflower and (**b**) Rape exines. P1-purified by Soxhlet extractions and an aqueous wash, and P2-purified by TBPH. Backscattered electron detector and accelerating voltage of 25 kV were used.

The results provided by the fluorescence microscopy were confirmed by ESEM, an electron microscopy method that does not require sample preparation (Fig. 2). For this purpose, we used TBPH-based P2-purification of sunflower and rape pollen, previously P1-purified by Soxhlet extractions. The pollen grains were mostly intact, and the intine wall was missing.

Note that we performed Atomic Force Microscopy (AFM) investigation as well. However, since the surface of the investigated pollen grain types was very uneven (height differences were greater than 1 µm within a region of 5 µm in diameter) our attempts were not successful.

#### Investigating the fluorescent coating of sporopollenin exine grains by fluorescence microscopy

**Figure 3:**
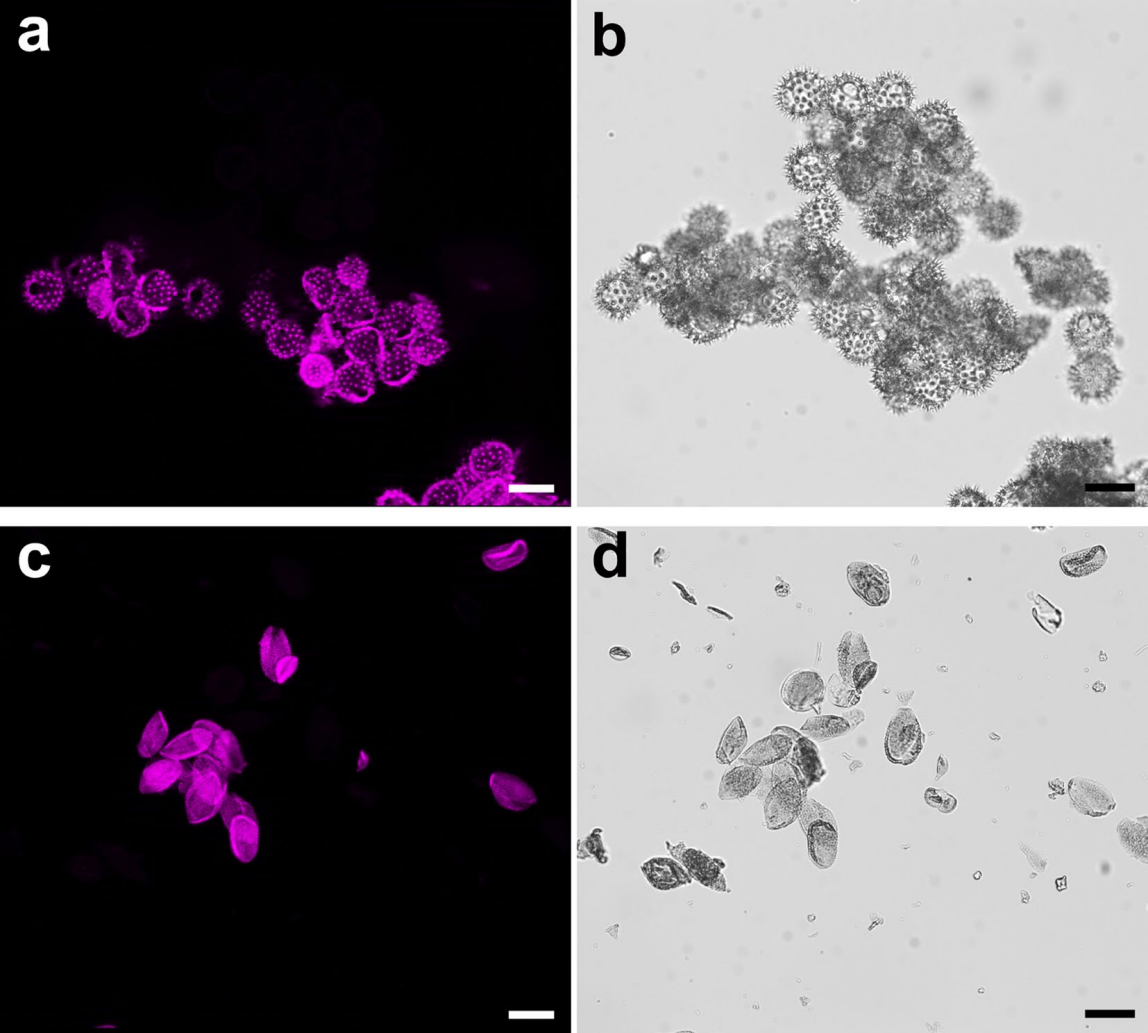
Fluorescence imaging of mixed samples of sporopollenin grains with and without fluorescent coating. Sunflower (**a**,**b**) and Rape (**c**,**d**) sporopollenin exine shells with and without RBITC coating. By using mCherry filtercube (**a**,**c**), only the coated grains can be shown, while the non-coated ones are glowing only weakly. Brightfield image (**b**–**d**) shows all of the grains.

In order to enable a comparison of the coated and non-coated sporopollenin exine particles, samples containing coated and noncoated sporopollenin exin grains were imaged using appropriate filter cubes (FITC for FITC coating, and mCherry for RBITC coating). Although sporopollenin exine variants have autofluorescence themselves, by using the appropriate filter cubes the difference in fluorescence of coated and noncoated particles becomes striking (Fig. 3).

#### Structure details disclosed by solid-state NMR spectroscopy

**Figure 4:**
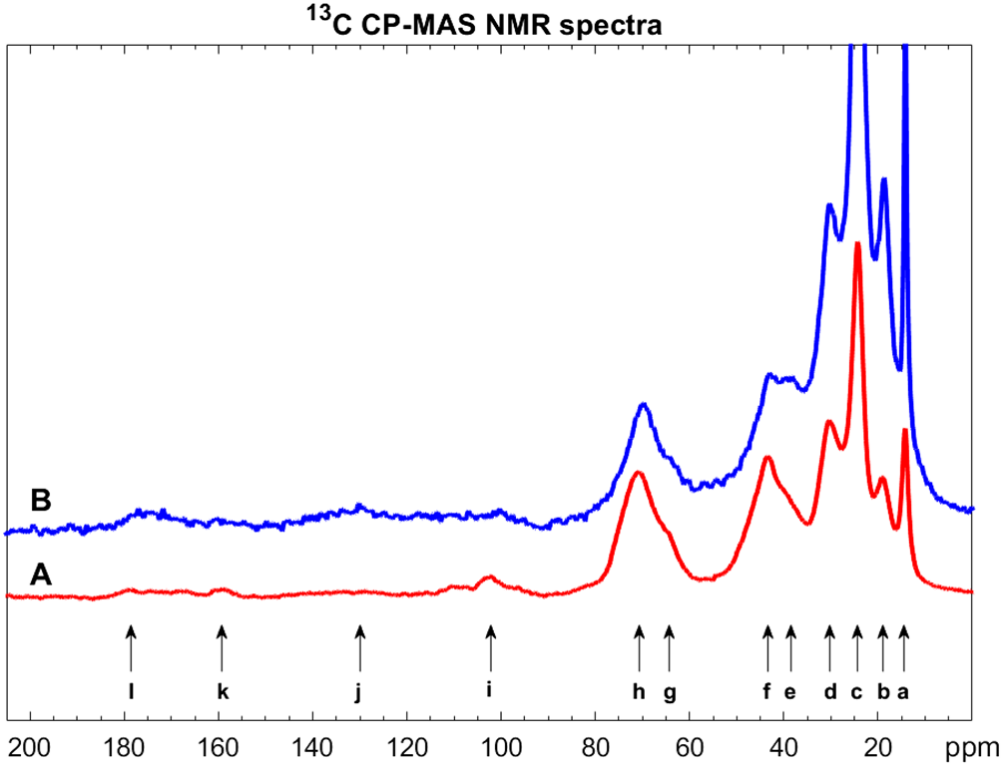
^13^C CP-MAS NMR spectra of sunflower (mark A, red curve) and rape (mark B, blue curve) sporopollenins treated by TBPH. The integral intensities of j, k aromatic carbon signals of p-coumaric groups and g, h O-aliphatic peaks were compared to each other.

**Figure 5:**
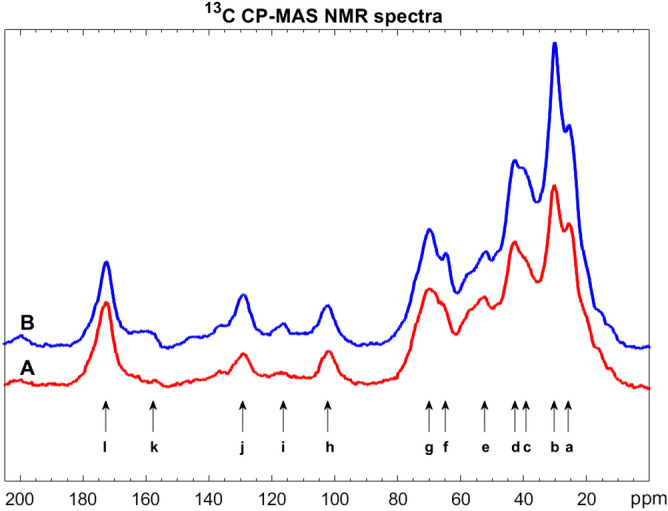
^13^C CP-MAS NMR spectra of sunflower (mark A, red curve) and rape (mark B, blue curve) sporopollenins treated by the enzyme cocktail. The integral intesities of i, j, k aromatic carbon signals of p-coumaric groups and e, f, g O-aliphatic peaks were compared to each other.

Samples of P2-purified sporopollenin grains, resulting from sunflower and rape, previously P1-purified by Soxhlet extractions and an aqueous wash, were investigated by ^13^C/^1^H CP-MAS NMR. The solid-state NMR spectra turned out to be similar to the previously published spectra of pine sporopollenin. The strongest peaks in our novel sunflower and rape sporopollenin spectra were identified by comparing them with the previously published pine spectrum^1^ (see Figs. 4, 5, as well as Tables 5, 6*.*).

**Table 5:** Assignation of ^13^C CP-MAS NMR spectra of sunflower (A) and rape (B) sporopollenins P2-purified by TBPH.

Notation	Chemical shift/ppm	Assignation
a	14.2	TBPA methyl
b	19.2	TBPA CH_2_-P
c	24.3	TBPA –CH_2_–CH_2_–
d	30.4	Aliphatic carbons
e	39.2	Aliphatic carbons
f	43.5	Aliphatic carbons
g	65.3	Oxygen-bearing carbons
h	70.6	Oxygen-bearing carbons
i	103	Acetal carbon
j	130	Broad aromatic carbon signals of *p*-coumaric groups
k	159	Broad aromatic carbon signals of *p*-coumaric groups
l	178	Terminal carboxylic group

**Table 6:** Assignation of ^13^C CP-MAS NMR spectra of sunflower (A) and rape (B) sporopollenins P2-purified by the enzyme cocktail.

Notation	Chemical shift/ppm	Assignation
a	25.6	Aliphatic carbons of C16 units
b	30.1	Aliphatic carbons of C16 units
c	40.5	Aliphatic carbons
d	42.8	Aliphatic carbons
e	52.0	Oxygen-bearing carbons
f	64.7	Oxygen-bearing carbons
g	70.0	Oxygen-bearing carbons
h	102.3	Acetal carbon
i	116	Broad aromatic carbon signals of *p*-coumaric groups
j	129	Broad aromatic carbon signals of *p*-coumaric groups
k	159	Broad aromatic carbon signals of *p*-coumaric groups
l	173	Terminal carboxylic group

First, we discuss the findings on the samples treated by TBPH (Fig. 4, Table 5). The 10–90 ppm aliphatic region contains several characteristic peaks. Both of our samples contain relatively sharp TBPH butyl signals (*a*, *b* and *c*) which are completely missing from the spectra of the same sporopollenins treated with the enzyme cocktail (see Table 6, where there are no peak data in the range of 14–24 ppm). The 30–50 ppm region shows aliphatic carbon signals of the sporopollenins in three broad, overlapping peaks (*d, e, f*). Peak *d* is identified as the signal of the C16 aliphatic unit. Peak *e* is significantly higher in the rape sample (B), than in the sunflower one (A). The 60–80 ppm range contains two overlapping signals of the oxygen-bearing, PVA-like carbons in both samples (signal *g* and *h*). The peak *i* belongs to the acetal group which forms a crosslink between the two PVA-like units and the 7-*O*-*p*-coumaroylated C16 aliphatic unit.

In order to reveal the differences between the structure of the rape and sunflower sporopollenins, we compared the integrals of the very broad aromatic carbon signals (*j* and *k*) of coumaric acid in the range of 106–165 to the area of *g-h* (O-aliphatic) peaks. The integral ratio is 15.7%:84.3% in the case of sunflower (A) and 35.2%:64.8% in the case of rape (B) spectrum which means that the sunflower sporopollenin contains much less coumaric acid groups than the rape sporopollenin. The signal *l* belongs to carbonyl carbons which are mostly the peaks of the terminal carboxylic groups and not of the alpha-pyrone because of the missing alpha-pyrone signals at 90 ppm.

The ^13^C/^1^H CP-MAS NMR spectra of enzymatically treated sunflower (A) and rape (B) sporopollenins are compared in Fig. 5 and Table 6*.* The sunflower and rape sporopollenin spectra are similar to each other, except for two higher aromatic peaks (*i* and *k*) in the rape sample. The aliphatic range of 20–90 ppm contains the carbon signals of the C16 units (*a*, *b*), and the CH_2_ signals of the different –CH_2_–CH(OX)–CH_2_– units (*c*, *d*). The 50–80 ppm range contains two broad, overlapping bands of the oxygen-bearing, PVA-like carbons in both samples (signal *e*, *f* and *g*). Peak *h* (at 102.3 ppm) is identified again as the signal of the acetal group, which forms a crosslink between the two PVA-like units and the 7-*O*-*p*-coumaroylated C16 aliphatic unit. By comparing the integrals of the aromatic carbon signals (*i*, *j, k*) of coumaric acid (106–165 ppm) to the area of *e, f, g* O-aliphatic peaks, it was found that the integral ratio is 37.6%:62.4% in the case of sunflower (A) and 44.3%:55.7% in the case of rape (B) spectrum. This result correlates with the quantitative analysis result of the TBPH treated samples.

However, the difference in the ratios measured for samples P2-purified by TBPH and the enzyme cocktail indicates that the treatment with TBPH more efficiently decreases the signals of the aromatic compounds (see i, j, k, and l signals in Fig. 5) of the pollen grains than the enzymatic process. The reason for this is unknown. We cannot exclude that the TBPH might remove some aromatic groups from certain regions of sporopollenin, without affecting the macroscopic structure of the exine. Further investigations using fractured exine shells and instrumental analytics are needed to clarify this issue.

#### NIR Raman spectroscopy confirms findings obtained by the solid-state NMR spectroscopy

**Figure 6:**
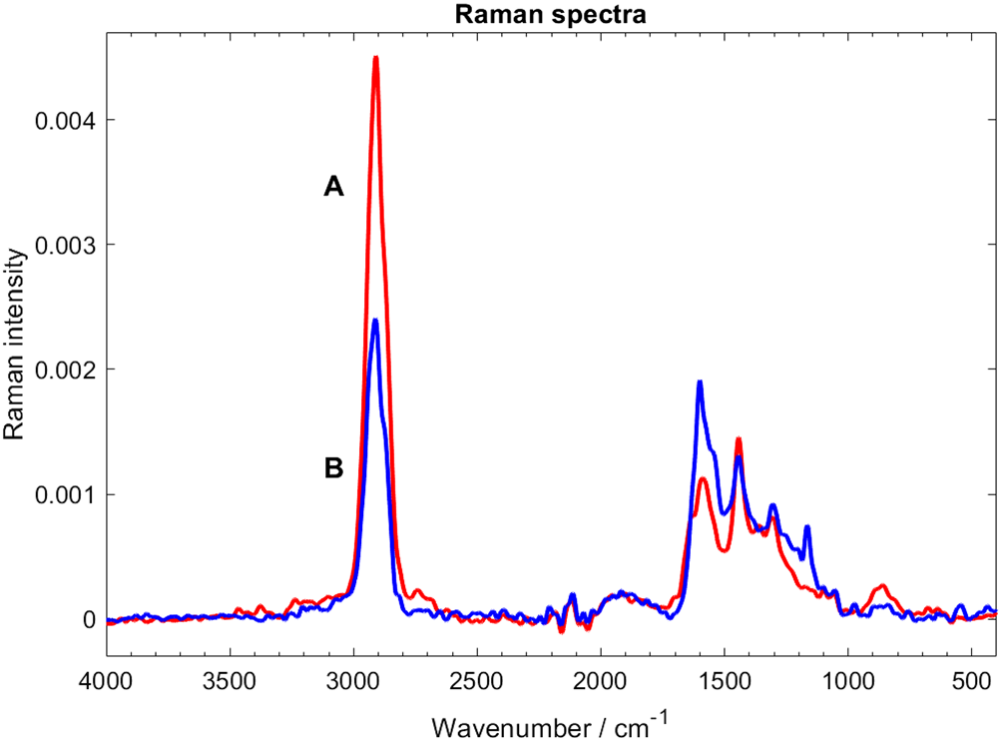
NIR FT-Raman spectra of sunflower (mark A, red curve) and rape (mark B, blue curve) sporopollenin P1-purified by Soxhlet extractions and P2-purified by TBPH. At 2910 cm^−1^ a strong aliphatic (mostly CH_2_ stretching), while around 1600 cm^−1^ aromatic bands are located. Note the difference in aliphatic to aromatic ratios for the two samples.

Near-infrared FT-Raman spectra of sporopollenin exine samples from sunflower and rape pollen P1-purified by Soxhlet extractions and an aqueous wash and subsequently P2-purified by TBPH are shown in Fig. 6*.* Raman bands arising from aliphatic chains are clearly identified at about 2910 cm^−1^ (mostly CH_2_ stretching) and 1444 cm^−1^ (CH_2_ scissoring), while aromatic ring vibrations show up at around 1600 cm^−1^, the rest of bands in the 1300–1000 cm^−1^ region may be related to C–C skeletal, aromatic in-plane C–H bending as well as stretching vibrations of C–O bonds. Beside evident similarities, the spectra show some characteristic differences, especially concerning the intensity ratios of Raman bands of aliphatic and aromatic components.

In concordance with the solid-sate NMR spectra, Raman spectroscopy results suggest that sunflower exine is richer in saturated, aliphatic components (higher-intensity of bands at 2910 and 1444 cm^−1^) than rape exine, where the 1600 cm^−1^ band, related to aromatic components is also stronger.

#### Autofluorescence spectra

Emission autofluorescence spectra for excitations at 300, 400 500 and 600 nm for sporopollenin exine samples P1-purified by Soxhlet extractions and an aqueous wash and P2-purified by TBPH, are shown in Supplementary Material Figure S1. As the excitation wavelength increased, the intensity of the autofluorescence decreased.

## Experimental procedures

### Bee pollen samples

Our core investigations were conducted on three types of bee pollen samples dried in laminar air flow at 40 °C. Rape had a purity of 100%, Sunflower of 93% (with 7% maize), and we examined a mixed pollen sample as well. The compositional analyses of the samples are listed in Supplementary Material Table S1.

Rape pollen was collected by a beekeeper in nort-western Hungary (Szigetköz region), while the sunflower by one in central Hungary (Fejér county). The mixed sample is a conglomerate of pollen samples collected in different Hungarian and Transylvanian regions in various seasons.

### Removing soluble compounds outside the cellulosic intine wall (P1 purification phase)

#### Soxhlet extractions

Soxhlet extractions of 100 g of bee pollen grains were performed in two types of extractors. In the thinner one, having an inner diameter of 5 cm, the height of the pollen column was 10 cm, the highest level of the solvent column above the pollen was 5 cm, and an extraction cycle took about 5 min. In the thicker one, with an inner diameter of 6.5 cm, the height of the pollen column was 6 cm, the highest level of the solvent column above the pollen was 10 cm, and a cycle took about 15 min.

The overall time of the extraction was 7 h. The volume of the solvents was 1 L (chloroform, isopropyl alcohol and methanol), while the extractors were equipped with 2 L round-bottomed flasks. The amount of the material extracted with the individual organic solvents was determined after rotational evaporation and subsequent drying at 60 °C at 5 mbar for 1 h.

Note that during extractions with chloroform and isopropyl alcohol the pollen grains stuck together while channels were formed within the material in the Soxhlet extractor. Therefore, in order to make the extraction more efficient, the pollen in the extractor was stirred several times.

After the extraction with methanol, the samples in the thin and thick extractors were combined, stirred for 3 h in 1 L of hot water, filtered on a glass filter (resulting in a clear flow, meaning that no pollen grains were lost) and dried at 60 °C at 5 mbar until weight constancy was achieved.

Finally, a last round of Soxhlet extraction with acetone was performed. This purification step did not remove any further material. The consistency of the resulting purified substance was a brittle solid, not a fine powder.

#### Washes with water and isopropyl alcohol

Aqueous washes were performed with hot (90–100 °C) as well as room temperature water, respectively. 100 g of bee pollen grains was mixed with 1 L of water and stirred for 3 h with a mechanical stirrer at 700 rpm.

Stirring in hot water was followed by filtering on a pre-heated glass filter. Heating of the filter (by rinsing it with hot water) was necessary to avoid the blocking of the filter by the waxy compounds. The pollen on the filter was rinsed with 300 mL of hot distilled water and dried in a vacuum desiccator over P_2_O_5_ until weight constancy was achieved.

Stirring in room temperature water was followed by decantation. The waxy material on the water surface was removed, while the rest was filtered. Again, the substance on the filter was rinsed with 300 mL of distilled water and dried in a vacuum desiccator over P_2_O_5_ until weight constancy was achieved. Note that the filtering in this case took several hours.

Finally, 30 g of dry material previously washed with hot water was homogenized in 300 mL of refluxing (80 °C) isopropyl alcohol; and 30 g of dry pollen previously washed with cold water was homogenized in 300 mL of room temperature isopropyl alcohol and the solutions were stirred for 3 h. Subsequently, they were filtered on a glass filter, rinsed with 100 mL of isopropyl alcohol having the same temperature, and dried in a vacuum desiccator over P_2_O_5_ until weight constancy was achieved. The resulting material was a fine powder.

#### Washes with isopropyl alcohol and Sodium Lauryl Sulfate aqueous solution

20 g of bee pollen grains was mixed with 200 mL of isopropyl alcohol, stirred for 3 h at room temperature, filtered on a glass filter, rinsed again with 50 mL of isopropyl alcohol and dried. Subsequently, the samples de-fatted in this way were stirred in 100 mL of 10 m/m % sodium lauryl sulfate solution for 3 h, washed five times with 100 mL of distilled water, filtered and dried in a vacuum desiccator over P_2_O_5_ until weight constancy was achieved.

In an alternative protocol, 150 g of bee pollen grains was mixed with 750 mL of 10 m/m % sodium lauryl sulfate solution and was let to stay for a day at room temperature. Subsequently it was centrifuged for 10 min at 5000 g, filled up with 500 mL of distilled water, and mixed. The procedure was repeated 6 times. Finally, the material was rinsed on a glass filter with 2 L of distilled water and 1 L of isopropyl alcohol and subsequently dried in a vacuum desiccator over P_2_O_5_ until weight constancy was achieved. However, this latter protocol led to the loss of some material while removing the supernatant after the centrifugation steps and turned out to be inadequate for measuring mass balances.

Both protocols yielded a fine powder.

### Dissolution of the cellulosic intine wall (P2 purification phase)

#### Hot 85% phosphoric acid

10 g of pollen P1-purified by Soxhlet extractions was immersed in 75 mL of 85% phosphoric acid at 90–95 °C for 5 h and was vigorously shaken every hour. Subsequently the material was vacuum-filtered, rinsed with 50 mL of phosphoric acid and 300 mL of distilled water. The time required for filtering varied between a couple of hours and one day. The treatment with strong acid resulted in a dark brown material. After rinsing with distilled water, the shade of the color became lighter. The resulting samples were dried in a desiccator over P_2_O_5_ until weight constancy was achieved.

#### Room temperature 85% phosphoric acid

10 g of pollen P1-purified by Soxhlet extractions was stirred in 75 mL of 85% phosphoric acid at room temperature for 5 days and was shaken several times every day. The procedure was the same as for the hot acid. Note that the vacuum filtering required significantly less time than in the case of the hot acid.

#### Hot 10% potassium hydroxide aqueous solution

10 g of pollen P1-purified by Soxhlet extractions was stirred in 50 mL of 10% potassium hydroxide (KOH) solution at 90–95 °C for 5 h and was vigorously shaken every hour. Subsequently, the material was filtered in vacuo and rinsed with 50 mL of potassium hydroxide solution, and 300 mL of distilled water. The time required for filtering varied between a couple of hours and 2 days. The procedure resulted in dark brown material. After rinsing with distilled water, the shade of the color became lighter. The washed samples were dried in a desiccator over P_2_O_5_ until weight constancy was achieved.

#### Room temperature 10% potassium hydroxide aqueous solution

5 g of pollen P1-purified by Soxhlet extractions was stirred in 200 mL of 10% potassium hydroxide solution at room temperature for 5 days and was shaken several times every day. The procedure was the same as for the hot alkaline. Note that in this case the vacuum filtering required significantly more time.

#### 2-Ethoxyethanol (Cellosolve)

5 g of pollen P1-purified by Soxhlet extractions was stirred in 50 mL of Cellosolve for 5 h, while being shaken every hour. Subsequently the material was filtered *in vacuo* and rinsed with 25 mL of Cellosolve and 50 mL of acetone. The washed samples were dried in a desiccator over P_2_O_5_ until weight constancy was achieved.

Note that the mass loss during the procedure using Cellosolve was either minimal, or, for sunflower pollen, even a slight mass increase occured. We speculate that the solvent could bind to the pollen grains. However, the consistency of the substance was unequivocally altered. The rigid, brittle material resulting from Soxhlet extractions was transformed into a fine powder.

#### Ionic liquid: 40% tetrabutylphosphonium hydroxide aqueous solution (TBPH)

0.5 g of pollen P1-purified by Soxhlet extraction was stirred with a magnetic stirrer in 5 mL of TBPH for 2 days at room temperature. Subsequently the material was filtered *in vacuo* and rinsed with 50 mL of distilled water. The washed samples were dried in a desiccator over P_2_O_5_ until weight constancy was achieved.

In order to decrease the damage of the pollen grains, stirring can be replaced by shaking, while vacuum filtering by centrifugation at 3000*g* for 10 min. In this case, the removal of the TBPH was implemented by two centrifugation and resuspension steps using distilled water. Drying was speeded up by subsequent resuspensions in isopropyl alcohol and hexane, followed by centrifugation.

#### Enzyme cocktails

In order to find the most efficient enzyme mix for dissolving the cellulose-rich intine wall, we tested a series of lignocellulose-degrading enzyme cocktails (Cellic CTec2, NS22086—Cellulase complex; NS22119—Enzyme complex; NS22002—Hemicellulase; and NS22118—β-glucosidase). All of these were provided by Novozymes A/S (Bagsvaerd, Denmark). A mixture of Cellic CTec2 (main activity: cellulase) and NS22118 (main activity: β-glucosidase) turned out to be the most effective for intine dissolution, and the results presented in the paper were implemented by a combination of these two cocktails. Filter paper activity of Cellic CTec2 was 142 FPU/mL, while the β-glucosidase activity of NS22118 was 318 IU/mL. Note that one unit of filter paper activity (FPU) is defined as the amount of released glucose (μmol) per minutes at 50 °C and pH 5 using Whatman No. 1 filter paper (Sigma) as substrate, and one unit of β-glucosidase activity (IU) is the amount of released glucose (μmol) per minutes at 50 °C and pH 5 using 4-nitrophenyl-β-d-glucopyranoside (Sigma) as substrate.

##### Dilute acidic pretreatment of soxhlet and water-isopropanol purified pollen grains

0.15 g of pollen samples P1-purified by several protocols were dispersed in 5 mL of 2% w/w sulfuric acid solution and treated for 1 h at 120 °C in a pressurized autoclave. After this procedure, the pollen grains were separated from the supernatant by centrifugation (6000*g*, 10 min) and washed two times with acetate buffer (100 mM, pH 5). Dilute acid pretreatments were carried out in triplicates.

##### Enzymatic hydrolysis

Pretreated and washed pollen samples were resuspended in 5 mL of enzyme solution containing 0.85 FPU of Cellic CTec2 and 0.29 IU of NS22118 in acetate buffer (100 mM, pH 5). The enzymatic hydrolysis was performed at 50 °C under continuous shaking for 24 h. To avoid microbial contamination, 20 mg of Thimerosal (Sigma) was added to 1 L of the enzyme solution. After enzymatic hydrolysis of the intine, the sporopollenin exine shells were separated from the supernatant by centrifugation (6000*g*, 10 min) and washed with distilled water two times. Dehydration took place in a drying chamber (50 °C) for 3 days. Enzymatic hydrolyses were carried out in triplicates.

### Chemical coating

The surface of the P1-, and P2-purified sporopollenin was coated with functionalized fluorescent dyes. Rhodamine B isothiocyanate and Fluorescein isothiocyanate (Sigma-Aldrich, Germany) were attached to the amino groups on the surface of the sporopollenin exine shells as described below. Our protocol can be regarded as a model for exine coating by further compounds with isothiocyanate or other reactive functional groups.

RBITC and FITC coating was implemented as follows. 5 mg of Rhodamine B isothiocyanate was added to a suspension of 10 mg of purified sporopollenin exine in 200 μL of DMF (*N*,*N*-dimethylformamide). The resulting suspension was shaken for 2 days. The pollen grains were separated by centrifugation and washed repeatedly with DMF until the color of the dye (RBITC of FITC) was not visible in the rinsing solution. Subsequently, the functionalized sample was washed twice with water, isopropyl alcohol, tetrahydrofuran and dried under vacuum. A similar procedure was applied for FITC coating.

### Instrumental analysis

#### Fluorescent microscopy

Integrity and shape of pollen grains was checked by brightfield microscopy.

Fluorescence microscopy was used, on one hand, to check the presence or absence of the cellulosic intine, and on the other hand, to examine the coating of the exine shells with fluorescent dyes. Images were taken at RT using an AxioImager Z1 epifluorescent microscope (Zeiss) equipped with an Apotome grid confocal unit and a HBO 100 mercury lamp, using AxioCam MRm camera and EC Plan-Neofluar 40 × NA = 0.75 or EC Plan-Neofluar 10 × NA = 0.3 air objectives (all Zeiss). Images of Calcofluor white, GFP, DsRed, and Dylight 488 fluorescence were acquired in AxioVision SE64 Rel. 4.9.1 (Zeiss) and processed in Photoshop CS3 Extended (Adobe).

In order to investigate the condition of the intine, calcofluor white^73–76^, a non-specific fluorescent stain was applied, which binds to structures containing cellulose, callose and chitin. Pollen grains were immersed either in 50% m/m aqueous solution of glycerol or in 1% m/m agarose gel, both containing a 1:1 v/v dilution of the calcofluor white solution (Sigma, 18909, Calcofluor white M2R 1 g/L, Evans blue 0.5 g/L). For fluorescent imaging, DAPI filtercube was used. Note that the sporopollenin exines show an autofluorescence as well (see Supplementary Material Figure S1). Therefore, our attention was primarily focused on the presence or absence of intine portions which protrude through the exine apertures.

To investigate RBITC and FITC coating, coated pollen grains were immersed in water or 50% m/m aqueous solution of glycerol. For the fluorescent imaging, mCherry and FITC filter cubes were used, respectively.

#### Solid-state NMR

The solid state ^13^C spectra were recorded at 125.77 MHz using cross-polarization (CP) and magic angle-spinning (MAS). Bruker Avance III 500 MHz spectrometer and Bruker 4 mm BB-1H MAS probe were used. Spectra were obtained with the following parameters: relaxation delay of 5 s, spectrum window of 80 kHz, sample spin-rate of 10 kHz. 2048 data points were acquired during 12.9 ms, with a SPINAL-64 1H decoupling sequence. A shaped Ramp 90–100% ^1^H pulse was used to get more efficient dipolar interaction during the CP. Spectra are referenced to an external sample of tetramethylsilane.

#### Raman spectroscopy

Sporopollenin variants show a strong autofluorescence when illuminated with ultraviolet or visible light. This makes it challenging to record high-quality Raman spectra using UV–VIS excitation^77–79^. However, when excited with NIR light, their autofluorescence is minimal. Therefore, we decided to perform FT-Raman spectroscopy employing near-infrared (1064 nm) excitation laser. Although near-infrared lasers are often used to record Raman spectra of biological samples showing strong fluorescence for the visible range, they have certain disadvantages. The lower energy of the near-infrared laser strongly reduces the efficiency of the Raman-scattering which may lead to decreased S/N ratio and potentially require much longer measurement time. Near-infrared lasers can also overheat the sample.

The FT-Raman spectra were recorded with a Bruker IFS55 Fourier-transform infrared (FTIR) spectrometer equipped with a FRA 106 Raman extension unit (Bruker Optik GmbH, Ettlingen, Germany), using a 500 mW 1064 nm Nd:YAG laser source operated at 100 mW laser power and a liquid nitrogen cooled Ge detector. The FT-Raman spectra of sporopollenin exine samples, placed in the standard solid sample holder of the instrument using a 180° back-scattering arrangement, were recorded at 4 cm^−1^ instrumental resolution in the 4000–100 cm^−1^ wavenumber (Raman-shift) range, and at least 2048 scans were accumulated. The raw spectra were baseline corrected for fluorescent background and a 19-point smoothing was applied for noise reduction.

#### Fluorescence analysis

Fluorescence spectra have been recorded by a Spex FluorMax fluorimeter. 20 mg of rape or sunflower exine purified by Soxhlet extractions and TBPH was dispersed in 5 mL of distilled water in a 10 × 10 mm quartz cuvette equipped with a magnetic stirrer. Monochromatic light of 300 nm, 400 nm, 500 nm and 600 nm was used to excite the sample. Emission spectra were recorded using wavelength windows of 10 nm, and integration time of 2 s (Figure S1a). We also recorded fluorescence spectra using a Raman microscope (Supplementary material Figure S1b).

#### Scanning electron microscopy

Scanning electron microscopy (Hitachi 2360 N, 25 keV, Robinson BSE detector) was used to check the integrity of pollen grains in low vacuum mode without any coating, after different treatments.

## Conclusions

By measuring mass balances, we quantitatively compared the efficiency of different protocols to obtain purified sporopollenin exine capsules of several bee pollen types.

The most effective protocol (that removed the most material) for P1 purification phase comprised a series of Soxhlet extractions applying solvents of growing polarity, and a final wash with hot water (residues for different types of bee pollen were between 19 and 27%). Note that washes with hot water and isopropyl alcohol led to almost as good results as the series of Soxhlet extractions.

In order to remove the cellulosic intine and the cellular debris, while keeping the sporopollenin exin grains intact, a cheap and simple enzymatic method has been developed. The key improvements of our method are (1) the acidic pre-treatment of the sample, which makes the enzymatic digestion more effective, and (2) the use of a novel enzyme cocktail for dissolving the intine coat.

Efficacy of various classical and novel P2 purification protocols has also been compared. Our novel enzymatic intine removal lead, for different types of bee pollen, to residue ratios between 18 and 28%, while the treatment with TBPH resulted in 12–32%. Note that the enzymatic treatment does not affect the exine. The TBPH also keeps the macroscopic sporopollenin exine structure intact, but its influence on the exine has not completely been understood.

Calcofluor white staining and fluorescence microscopy seemed to be the most robust method to examine the presence or absence of the cellulosic intine. Instrumental analytical methods like VCD (vibrational circular dichroism) or FTIR-ATR (Fourier transform infrared spectroscopy—attenuated total reflection) did not lead to conclusive results.

An exceptionally interesting result was provided by the solid-state NMR and NIR Raman spectroscopy. Both methods confirmed that the ratio of the aromatic to O-aliphatic compounds is higher in rape than in sunflower exine.

We implemented a novel chemical coating of sporopollenin exine particles by fluorescent dyes with an isothiocyanate functional group. This result expands possible applications of sporopollenin exine grains. Our novel, coated, strongly fluorescent exine grains can be applied, among others, for biomedical research like investigating persorption or examining dissolution of sporopollenin grains in the blood.

## Supplementary Information

Supplementary Information.
