# Supplementary material for: Facile isolation and analysis of sporopollenin exine from bee pollen
Source: Sci Rep. 2021 May 11;11:9952. doi: 10.1038/s41598-021-87619-8 (PMC8113464; doi:10.1038/s41598-021-87619-8)
Supplement: Supplementary file 1 — Supplementary Information. [file 41598_2021_87619_MOESM1_ESM.docx]

**Supplementary Material**

**Facile isolation and analysis of sporopollenin exine from bee pollen**

Kristóf Hegedüs^1^, Csaba Fehér^2^, István Jalsovszky^3^, Zoltán Kristóf^4^, János Rohonczy^5^, Elemér Vass^3^, Attila Farkas^6^, Tamás Csizmadia^7^, Gernot Friedbacher^8^ and Peter Hantz^10,3,9^**^#^**

^1^Institute of Organic Chemistry, Research Centre for Natural Sciences, Magyar Tudósok Körútja 2, H-1117 Budapest, Hungary

^2^ Budapest University of Technology and Economics, Department of Applied Biotechnology and Food Science, Budapest, Szent Gellért tér 4, H-1111 Budapest, Hungary

^3^ Eötvös Loránd University, Department of Organic Chemistry, Pázmány Péter sétány 1/A, H-1117 Budapest, Hungary

^4^ Eötvös Loránd University, Department of Plant Anatomy, Pázmány Péter sétány 1/A, H-1117 Budapest, Hungary

^5^ Eötvös Loránd University, Department of Inorganic Chemistry, Pázmány Péter sétány 1/A, H-1117 Budapest, Hungary

^6^ Budapest University of Technology and Economics, Department of Organic Chemistry and Technology, Budapest, Budafoki út 8, H-1111 Budapest, Hungary

^7^ Eötvös Loránd University, Department of Anatomy, Cell and Developmental Biology, Pázmány Péter sétány 1/C, H-1117 Budapest, Hungary

^8^ Vienna University of Technology, Institute of Chemical Technologies and Analytics, Getreidemarkt 9, A-1060 Wien, Austria

^9^ Fibervar Llc., Str. Bolintineanu Nr. 20, RO-400062 Cluj/Kolozsvár, Romania

^10^ Centre for Ecological Research, Karolina út 29, H-1113 Budapest, Hungary

**^#^** Corresponding author

Correspondence: hantz@general.elte.hu

| Sunflower | Rape | Mixed |
| --- | --- | --- |
| 93% Sunflower (*Helianthus annuus*)  7% Maize (*Zea mays*) | 100% Rape (*Brassica napus*)  /some grains damaged/ | No predominant pollen (more than 45%)  24% Sunflower (*Helianthus annuus*)  14% Willow (*Salix sp*.)  13% Rape (*Brassica napus*)  12% Meadowsweet (*Filipendula ulmaria*)  7% Brown knapweed (*Centaurea jacea*)  6% Thistle (*Carduus sp*.)  6% Trefoil (*Trifolium sp*.)  5% Dandelion (*Taraxacum officinale*)  5% Purple loosestrife (*Lythrum salicaria*)  4% Apple (*Malus domestica*)  2% Plum (*Prunus domestica*)  2% Marguerite (*Leucanthemum vulgare*)  Other pollen (less than 1%):  Bearbine *(Convolvulus arvensis)*  Oak *(Quercus sp*.)  Red poppy *(Papaver rhoeas)* |

***SM Table 1***

Palinology analysis of the used pure and mixed bee pollen samples. Palynological analyses were carried out by the standardized methods of the International Honey Commission from 2004, that is the revised method of Louveaux-Maurizio-Vorwohl from 1978. Identification of the pollen types can be achieved by observing the main structural properties like exine structure, size, outline, and aperture type. The investigations were carried out using light microscopy (400X magnification) by embedding the grains in glycerine-fuchsine jelly and glycerine jelly, respectively.


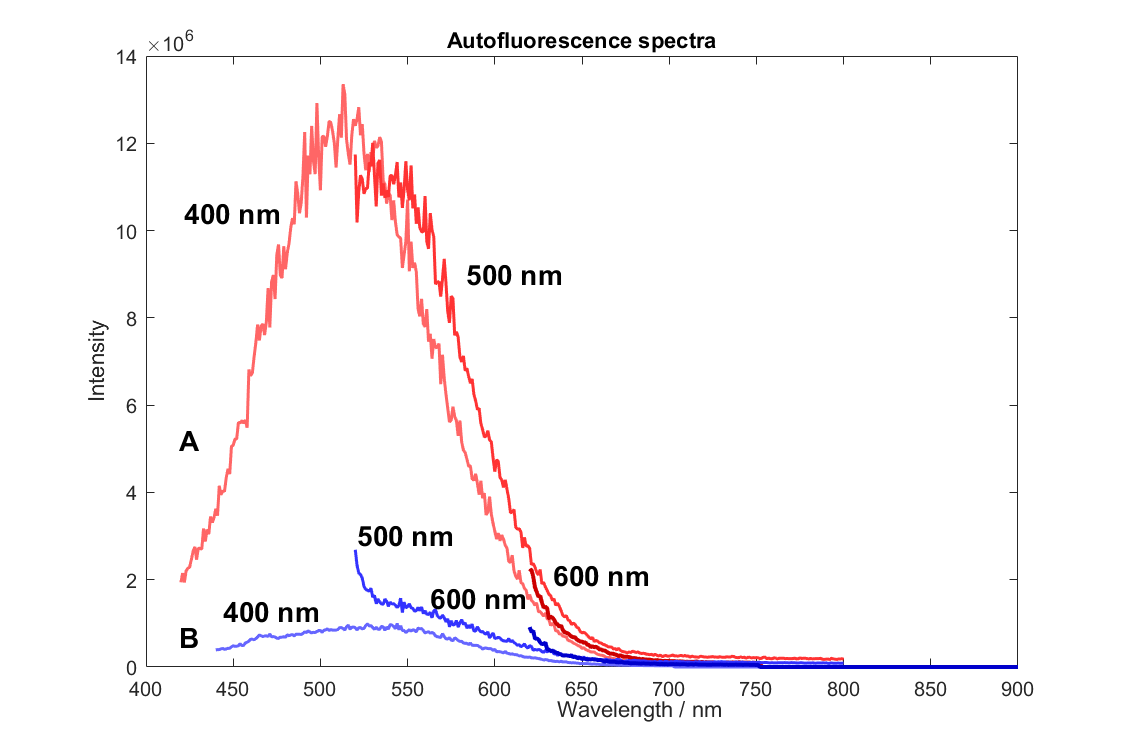


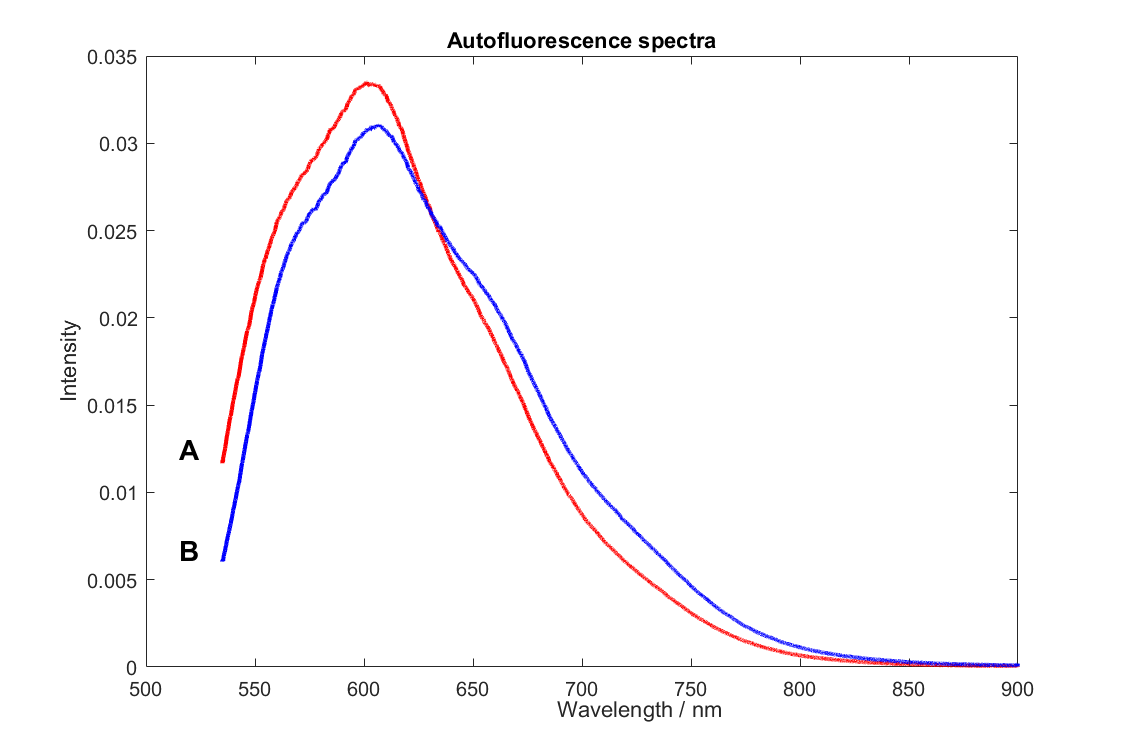
(a.)

(b.)

***SM Figure 1***

*Autofluorescence spectra of sporopollenin exines. Curve “A” denotes Sunflower, while “B” Rape exine spectra.*

*(a.) Spectra recorded by a Spex Fluor Max fluorimeter, excitation wavelength 400, 500, and 600 nm.*

*(b.) Spectra recorded by a Horiba Jobin-Yvon LabRAM Raman microscope coupled with an Olympus BX-40 optical microscope, excitation wavelength 532 nm.*


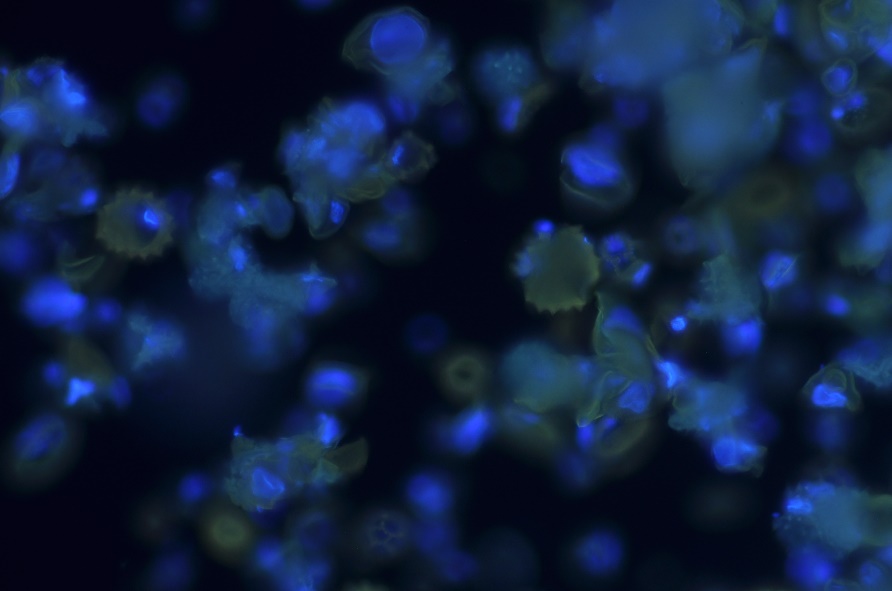


***SM Figure 2***

*Pollen grains after P1-purification by Soxhlet extractions and an aqueous was, and P2-purification with KOH. Some exines were destroyed, and the intine removal was incomplete.*
